# A survey of feline behavioral problems in Tehran

**Published:** 2015-06-15

**Authors:** Naqa Tamimi, Abdolali Malmasi, Aniseh Talebi, Fatemeh Tamimi, Atoosa Amini

**Affiliations:** 1 *Department of Internal Medicine, Faculty of Veterinary Medicine, University of Tehran, Tehran, Iran; *; 2 *Department of Statistics, School of Mathematics, Statistics and Computer Sciences, College of Science, University of Tehran, Tehran, Iran.*

**Keywords:** Behavioral problem, Cat, Tehran

## Abstract

Behavioral problems in cats have drawn more attention in recent years since they affect the cat-owner relationship. This study was designed to study the rate of cats with undesirable behaviors according to their owners. Frequency of behavioral problems in 167 cats attending Small Animal Hospital, Faculty of Veterinary Medicine, University of Tehran, was evaluated using a questionnaire. According to the results, 94.6% of owners reported that their cats have exhibited at least one undesirable behavior. Fearfulness, attention seeking, aggression towards other cats/people, scratching, and elimination problems were the most prevalent behavioral complaints reported by the owners; whereas obsessive behaviors were the least common behavioral complaints. In addition, data analysis suggested that age, breed, outdoor access, owner reaction towards the behavior and the cat’s interaction with other cats/people might have been associated with the development of some behavioral problems in cats. Considerable rate of undesirable behaviors in domestic cats in Iran is important enough to highlight the significance of veterinary intervention.

## Introduction

Companion cats have increased in number and even surpassed the number of dogs in some countries.^1^^-^^3^ Because cats are easier to being taken care of than dogs,^4^^,^^5^ many people prefer to keep cats.

Behavioral problem by definition is any behavior shown by an animal (in this context a cat) that is unacceptable to the owner. It includes behaviors such as marking which is a normal feline behaviors, yet, in domestic environment, they become undesirable.^6^ Scratching and predatory behavior are other examples of normal but nuisance behaviors of companion cats.^7^

Behavioral problems are very important because they are one of the most common reasons for the relinquishment of cats.^8^ They have been known to be the reason behind the death and relinquishment of more pets per year than infectious, neoplastic, and metabolic diseases altogether.^9^ A study indicated that 38.0% of cats were relinquished to a shelter because of their behavioral problems, the most common was aggression towards other cats.^10^ Among all behavioral problems aggression, scratching and inappropriate elimination have been the most common behavioral problems in cats, and have been associated with their relinquishment to shelters.^11^^,^^12^

From a public health point of view behavioral problems are also important because although the percentage of aggression problems is higher towards other cats than towards people, a high number of aggressive episodes are still directed towards people. There is no need to over emphasize the importance of physical and psychological effects of the attacks, especially when the targets are immune-compromised people.^13^^,^^14^ In addition, behavioral problems can have negative implications on the animal's welfare, specifically when they are the result of anxiety.^15^ Having said all that about the significance of behavioral problems in domestic cats we should further highlight the lack of professional veterinary behavior practice in Iran.

In order to use the best preventive measures, epidemiological studies can provide us with general prevalence of behavioral problems in domestic cats and help understand their associated risk factors. While veterinary behaviorists have published detailed studies regarding small animal behavior around the globe^16^^-^^18^ not many studies have been undertaken in the Middle-East including Iran.

The objectives in this preliminary survey were to describe the most common behavioral problems reported by owners in a feline population who attended the Small Animal Hospital, Faculty of Veterinary Medicine, University of Tehran, Iran, during a one year period and to identify the association of some characteristics of cats with development of the given behavioral problems.

**Study design and subjects.** A convenient sample was used from the caseload of the Small Animal Hospital, Faculty of Veterinary Medicine, University of Tehran, Iran. From June 2008 to June 2009, owners who had referred their cats to this hospital, for routine checkups were interviewed and completed a questionnaire. Except for kittens under the age of four months the remaining 167 cats were included in this survey.

**Data collection.** A questionnaire was specifically developed for this survey which included 40 behavioural questions. The owners’ answers were collected during an interview completing the questionnaire. Basic information such as age, sex, breed, outdoor access, its interaction with people other than family members and other animals (dogs, cats, ect.) were also recorded. Owners were also able to fully describe the cats’ behavior in open-ended questions at the end of the questionnaire. The frequency of the following behavioral problems was evaluated: Aggression towards people and other cats, elimination problems (house soiling, marking), scratching in-appropriate objects, fearfulness, attention seeking, excessive grooming, chasing small animals, hiding, vocalization in owner's absence, obsessive behaviors and eating fiber material. Brief descriptions of behavioral complaints reported by cat owners evaluated in this survey are shown in Table 1. The frequency of these behavioral problems was assessed on the basis of owners' answers. Further questions regarding the owner's reaction towards the unwanted behavior and his/her intention to seek help for the given problem were also asked.

**Table 1:** Brief description of behavioral complaints evaluated in the current survey

**Behavioral problem**	**Definition**
**Fearfulness**	Showing any sign of fear towards different targets
**Attention seeking**	The cat tries to attract its owner's attention using different methods
**Aggression towards other cats**	Expressing aggressive behaviors towards other cats, from mild to serious (e.g. biting)
**Aggression towards people**	Expressing aggressive behaviors towards people (familiar or unfamiliar), from mild to serious (e.g. biting)
**Scratching inappropriate objects**	Scratching inappropriate objects in and around the house
**Inappropriate elimination:** **Marking** **House soiling**	Elimination in inappropriate spots in either of the two below: Spraying small amount of urine on specific spots specially vertical objects with the typical figure of marking and retaining normal use of litter box Complete evacuation of the bladder or bowels in inappropriate spots with loss of use of litter box
**Vocalization in owner absence**	The cat vocalizes when its owner is not around
**Chasing small animals**	The cat chases small animals like squirrels and birds if it gets the chance
**Hiding**	The cat tries to avoid interaction with its owner
**Excessive grooming**	The cat spends a significant part of the day grooming itself
**Obsessive behaviors**	Expressing out of context behaviors that interfere with the cat’s normal life
**Eating fiber material**	Tending to eat fiber material

**Statistical Analysis. **The Pearson Chi-square test was used to evaluate the correlation between occurrence of behavioral problem and interaction with other animals and people, outdoor access, owner reaction towards the behavior, age, breed, and sex of the cat. Confidence limit of 99.00% was used for chi-square test. A value of *p *< 0.05 was considered significant for the analysis. The statistical analyses were computed using SPSS (Version 15; SPSS Inc., Chicago, USA).

## Results

Basic information of 167 cats included in this survey is shown in Table 2. The average age of the cats was 1.9 years and the average age at which the cats were obtained was 2.6 months.

**Table 2:** Main characteristics of 167 cats included in this survey

**Characteristic **	**Number of cats**	**Percentage**
***Sex*** Male Castrated Intact Female Neutered Intact	77 15 62 90 24 66	9.0% 37.2% 14.3% 39.5%
***Breed*** Domestic short hair Persian Other	145 20 2	86.8% 12.0% 1.2%
***Age (Month)*** Less than 6 Between 6 to 11 Between 12 to 20 Between 21 to 35 More than 36	21 46 33 27 40	12.6% 27.5% 19.8% 16.2% 23.9%
***Outdoor access *** Yes No	53 114	31.7% 68.3%
***Interaction*** People* Cats† None	27 62 78	16.2% 37.1% 46.7%
***Owner reaction towards behavior***		
Punishment Ignoring/Distracting Comforting Inconsistent	53 38 32 44	31.7% 22.8% 19.2% 26.3%

* People other than family members;

† Multi-cats household.

Assessing the results, 94.6% of the cats were reported to have at least one behavioral problem. The mean number of behavioral problems in cats assessed in this survey was 2.71 (Median: 2). Only 19 (11.4%) of owners tended to take action in order to solve their cats' behavioral problem. The frequencies of behavioral problems reported in these cats are shown in Table 3.

**Table 3:** Frequencies of behavioral problems reported in 167 domestic cats

**Behavioral problem**		**Number of cats**	**Percentage**
**Fearfulness** † Fear of dogs Fear of people Fear of cats Other fears		76 47 45 6 2	45.5%
**Attention seeking**		62	37.1%
**Aggression towards other cats**		55	32.9%
**Aggression towards people**		53	31.7%
**Scratching inappropriate objects **	51		30.5%
**Inappropriate elimination** † House soiling Marking		51 46 7	30.5%
**Vocalization in owner absence**		25	15.0%
**Chasing small animals**		25	15.0%
**Hiding**		23	13.8%
**Excessive grooming**		19	11.4%
**Obsessive behaviors** Psychogenic alopecia Staring		15 8 7	9.0%
**Eating fiber material**		8	4.8%

† Some cats may have multiple problem types, so the total sum may surpass the cat’s number.

Data analysis suggested an association between age groups and attention seeking indicating that older cats were more likely to seek attention from their owners (*p* = 0.019). In addition, attention seeking appeared to have a correlation with the cats' sex as the intact females showed less tendency for attention seeking than other cats (*p* = 0.013). Further, the cats' breed was associated with aggression towards people (*p* = 0.013) and house soiling in this study (*p* = 0.044). In other words, Persian cats were significantly reported to have more house soiling problems than domestic short hair (DSH) ones, while they represented fewer tendencies for aggressive behaviors towards people than DSH cats according to their owners.

Access to outdoor was not associated with the presence of behavioral problems except for inappropriate elimination (*p* = 0.018) with the cats having access to outdoor being reported with more elimination problems than their counter-parts. Also, interaction with other cats and people was correlated with inappropriate scratching in cats' suggesting that cats with less interaction with people showed more tendency for scratching inappropriate objects (*p* = 0.027). Finally, cats that were punished for the food stealing behavior were significantly more likely to show this behavior (*p* = 0.002). This is when owners’ reaction towards the behavioral problem was not correlated to other behaviors.

## Discussion

According to the current survey 94.6% of cats were reported to have at least one type of undesirable behavior according to their owners with the average number of being 2.7 for each cat. This rate is considerably high but similar to what was reported by Edwards *et al*. in 2002 who reported that 100% of cats attended the Universidad Nacional Autónoma de México Veterinary Hospital were reported to have at least one behavioral problem.^17^ On the other hand, our result (94.6%) was substantially different from the 47.9% rate reported by another study in 2011 targeting a similar population in Tehran.^19^

Most owners seem to cope with the behavioral problems of their cats instead of seeking help from a professional since according to the results only 11.4% of owners tended to take action in order to solve their cats’ behavioral problem. Another explanation for this rate of owners’ ignorance towards behavioral problems of their cats in Iran is that these problems may not seem serious to owners or that they are unaware of how easily some of them can be resolved.

Fearfulness (45.5%) and attention seeking (37.0%) were the most common owner complaints according to the current survey. Aggression towards other cats (32.9%), aggression towards people (31.7%), inappropriate elimination (30.5%) and scratching (30.5%) were also common and were reported with similar rates in the current survey (Table 3). Our results seem different from the results of another study run in Mexico that reported scratching and destructive behaviors as the most common behavioral problems in cats.^17^ Geographic differences and owner perception can possibly explain this difference.

The rates of behavioral problems can vary widely in different locations and studies. For instance, data that are obtained through a questionnaire to the general practitioners differ from those obtained through a consultation in a referral service.^20^ The fact that owners might not refer their cats for specific behavioral reasons until some irritating behaviors (such as aggression or elimination problems) happen should also be kept in mind. This might justify the difference between results of the current survey and the results obtained in referral practices such as reports of aggression and elimination problems as the most common behavioral problems by Amat *et al*. who found similar results to other referral practices.^11^^,^^21^^-^^24^ The inappropriate elimination was a behavior reported with a close rate in our survey (30.5%) to that of the association of pet behavior counselors^23^ and Amat *et al*. (39.0%) which appears to be close to the rate obtained and the difference seems only in the ranking of the problem.^11^

Aggressive behaviors, scratching and inappropriate elimination which were associated with the relinquish-ment of cats according to Souza *et al.* were noted to be common in our survey.^12^ Human directed aggression, a potentially serious behavior problem in cats, was reported in 31.7% of the cats in our survey (Table 3) while other studies cited different rates (47.0%) for this behavior in cats. The main reason could be the different populations and that their reports reflect referral practices.^11^^,^^25^

Feline fear can be an important issue in the cat behavior practice; a fact that was reflected in the results of this survey as fearfulness was reported with the highest rate (45.5%). It is less likely for a cat to be presented for its fearfulness, since this behavior seems to be tolerated by most owners. This fact was shown by Amat *et al*., when they reported as few as six cats out of 336 cats referred for their fear or phobic problems.^11^ Is it only the source of information or are there other factors (such as the owner's expectation) involved, is open to debate because Naderi *et al.* reported low rate of 3.6% in a similar population of cats in Tehran.^19^ This high rate of fearfulness in domestic cats in our study can pose this query that those factors could contribute to this problem necessitating further assessment of this behavior.

Analyzing the results of this study suggests that outdoor access could be a risk factor for elimination problem while limited interaction of cats with people could be associated with more inappropriate scratching in these animals; therefore, it appears that having interaction with other people can have a good impact on reducing the cat's scratching behavior. As cited by other studies, Persian cats were reported to have more elimination problems.^11^ They also showed less aggressive behaviors towards people than their DSH counterparts. Takeuchi and Morio also found similar results in 2009 in that Persian cats showed mild aggression in comparison with other breeds.^26^ Cats that were reported for stealing food in our survey presented this problem more whenever they were punished for this behavior. Different training methods and the way owners react to their cats’ behaviors are important elements and it is suspected that specific training methods could be associated with the performance of behaviors deemed problematic by owners.^18^ Although no age predilection has been noted in attention seeking behavior in small animals in another study,^7^ this behavior was seen more frequently in older cats in our survey. Castration rate was low (Table 2) in our study therefore, it was not assessed separately as a risk factor for behavioral complaints in cats.

It should be noted that the term behavioral problem is very subjective and what may be considered a “behavioral problem” by one owner may be regarded as relatively “normal” by another. Apart from the terminology of cat behavioral problem, however, the fact remains that cats are frequently relinquished because their owners consider their behavior undesirable.^27^

In conclusion, the high number of cats reported with undesirable behaviors warrants that professional practitioners are needed to help overcome this problem by increasing awareness and educating owners and veterinarians. Recognizing some factors that can potentially affect the occurrence of behavioral problems in cats can also be a valuable contribution to this novel field in Iran. Large scale and more comprehensive studies are needed to further evaluate the findings obtained by this preliminary survey.
